# Return to work rate of individuals after cardiac rehabilitation and the demographic and impairment factors that influence return to work in the Western Cape, South Africa

**DOI:** 10.12688/f1000research.129263.1

**Published:** 2023-07-27

**Authors:** Zakeera Ganie, Shaheed Soeker, Anthea Rhoda

**Affiliations:** 1 Department of Occupational Therapy, University of the Western Cape, Bellville, Western Cape, 7535, South Africa

**Keywords:** cardiovascular disease, cardiac rehabilitation, vocational rehabilitation, return to work

## Abstract

**Background:** Cardiovascular disease commonly affects individuals within the working age group, often resulting in unemployment, particularly in low- to middle-income countries. The purpose of the study was to determine the return-to-work rate of individuals with cardiovascular disease after cardiac rehabilitation (CR) and the impact of impairment and socio-demographics on the individual’s ability to RTW.

**Methods:** A cross-sectional survey, namely the Work Rehabilitation Questionnaire was used to gather the information. The IBM SPSS software (version 25) was used to manage the statistical analysis. Individuals who completed a cardiac rehabilitation program between the ages of 18 and 64 years made up a sample of 63 potential research participants.

**Results:** The return-to-work (RTW) rate reported that only 30 (47.6%) of the participants successfully returned to work after CR and 33 (52.4%) of participants did not RTW. The results also indicated that the older the individual and the higher the degree of impairment experienced, the less likely RTW would occur.

**Conclusion:** The study concluded that only 47.6 % of individuals completing cardiac rehabilitation returned to work. Being older and the degree of impairment impacts on one’s ability to return to work. Factors such as the age and level of functional impairment of the individual with cardiovascular disease must be addressed more aggressively in cardiac rehabilitation programs, particularly if the goal of the individual with cardiovascular disease is to RTW.

## Introduction

The impact that cardiovascular disease (CVD) has on the economy is said to be substantial as it is increasingly affecting individuals in their productive years of life. ^1^ This burden of disease heeds calls to a more comprehensive approach for prevention and management of CVD, particularly in low- to middle-income countries such as South Africa. ^1^ Upon investigating the impact of evidence-based cardiac rehabilitation (CR) programs it was found that participation in such a preventative program does have an impact on decreasing CVD mortality and re-hospitalisation, as well as having an impact on an individual’s ability to return to work (RTW). ^2^ A prospective study conducted with 83 patients of working age during their CR found that job satisfaction and a positively perceived work environment resulted in early RTW after cardiac intervention and may have economic benefits and improve quality of life. ^3^

Slebus *et al.* ^4^ further explored facilitating factors for RTW and identified the following: no signs and symptoms of the disease, work contentment, positive relationships at work, ability to participate in work activities, information from the doctor that encouraged RTW, medical care was working well, family relationships were positive, financial motivation and intrinsic motivation to work. Similar results from a qualitative study of perpetuating factors for long-term sick leave and promoting factors for RTW, found that illness perceptions and self-efficacy expectations can be promoting factors for RTW. ^5^ Furthermore, financial obligations and culture are also contributing factors of RTW according to a study that reviewed the employment status after myocardial infarction (MI) among men. These men stated that they did not want to take advantage of their wives earning an income and that it was their responsibility as the breadwinner to provide for their families. ^6^

In a Danish cohort study examining RTW after hospitalisation for heart failure, the authors speculate that a lack of RTW was due to functional limitations as a result of the disease as well as the psychological effects of having the heart failure diagnosis. ^7^ Kearney *et al.* ^8^ also observed that fatigue was most frequently reported as a barrier to RTW, followed by mild cognitive impairment such as impaired memory and cognitive processing. Similarly, a study with patients who had implantable cardioverter defibrillators (ICDs) estimated that 75% of patients experience mild to severe short-term cognitive impairment, with roughly 33% of these expected to be prolonged at six months. ^9^

Patients suffering from anxiety or emotional distress following the cardiac incident have been found to have increased difficulty in lifestyle modification and less likely to complete CR. ^10^ This could have a negative impact on the individual’s prospects of RTW and successfully maintaining employment. Similarly, in a prospective cohort study examining the associations between depressive episodes and anxiety disorder with RTW after MI at three and six months found that the presence of a depressive episode and anxiety disorder during the first three months after the MI was a significant predictor of not returning to work by 12 months. ^11^

Kearney *et al.* ^8^ also found that patients in occupations such as labourers, tradesman and in the transport industry were less likely to RTW as their jobs were labour-intensive and that their illness had a greater impact on their ability to perform their work tasks. Add the significance of the study- relate the significance to the aim of the study. The significance of the current study was to determine the demographic factors that are associated with return to work (RTW) for individuals with cardiac conditions. The latter information would determine the RTW rate of individuals after they have completed a cardiac rehabilitation program. Furthermore, the study would determine which specific factors influence RTW for individuals with cardiac conditions after the completion of a cardiac rehabilitation program.

### Aim and objectives

• To determine the RTW rate of individuals with CVD after a cardiac incident
• To determine the impact of impairment and socio-demographic factors on an individual’s ability to RTW after a cardiac incident

## Methods

### Participant recruitment

All participants were patients of a district hospital, who were admitted due to a cardiac incident between 2017 and 2018. Each participant was referred to CR as an outpatient. The sampling technique utilised in this study was convenience sampling. A convenient sample was utilised at the data collection site. The advantage of using this sampling technique is that it is inexpensive, and results can be sorted quickly. However, there is a risk of the sample not being representative. ^12^ This study yielded a sample of 63 participants. Cross-sectional surveys in form of the Work Rehabilitation Questionnaire (WORQ) were used to achieve the objective of determining the RTW rate after participation in the CR program. The WORQ is a validated and reliable tool used to measure functioning in the vocational rehabilitation setting. ^13^

Furthermore, the survey provided information regarding gender, age, marital status, education level and an impairment score. After a trial that took place in two months, the researcher collected data over a one-year period. Data was collected through face-to-face interviews and telephonically. The IBM SPSS software was used to conduct measurements of central tendency, frequencies and percentages of variables.

### Participants

**Description of the study population**

The population for this study comprised individuals attending the CR program at the research site in the Western Cape. The district hospital only accommodates patients residing in the southern suburbs, who have been admitted to the hospital as inpatients. The inclusion criteria for participation was an age of 18-64 years and active employment prior to the cardiac incident. Seventy-seven copies of the questionnaire were administered, however only 63 surveys were accepted for analysis. Fourteen surveys were incomplete and therefore voided.

**Demographics and clinical characteristics of the study sample**

The study sample comprised 27 female and 36 male participants, with a mean age of 54 years and a standard deviation of 5.95. Most (60.3%) of the participants in the sample were in the 51-60 year age group. Forty-two of the participants were married, 12 divorced, 7 never married, 1 widowed and 1 was cohabiting. The majority (73%) of participants had a secondary school education. Forty-two participants completed secondary school, with 8 graduates from college/university, 2 had completed post-graduate studies and 7 primary school only. All the participants had experienced a MI between 2016 and 2018 and received medical and therapeutic intervention. Nineteen participants were receiving government support in the form of social grants ( Table 1).

**Table 1.** Demographics of the participants.

Variable	n (63)	%
**Gender**		
Female	27	43
Male	36	57
**Age (years)**		
≤40	1	1.6
41-50	16	25.7
51-60	38	60.3
≤61	8	12.7
**Marital status**		
Single	7	11
Married	42	67
Divorced	12	19
Widowed	1	1.6
Co-habitin	1	1.6
**Education level**		
Primary school	7	11
Secondary School	46	73
Tertiary	8	13
Postgraduate	2	3
**Received medical or therapeutic intervention**		
Yes	63	100
No	0	0
**Government support**		
Yes	19	30
No	44	70

**Description of cardiac rehabilitation program**

The individuals diagnosed with a MI were provided with a pamphlet with information on MI, medication, exercise and ADLs that are safe to do. All patients with MI are usually referred for CR at the district hospital. The CR program at the hospital is an education-based program, aimed at lifestyle modification. It consists of weekly education sessions that are arranged in a three-week cycle, that focuses on the use of medication, healthy eating, graded exercise that can be done at home and participation in activities of daily living, as well as psychological aspects that can arise after a cardiac incident. The program is facilitated by the occupational therapist and physiotherapist. Talks by consultant physicians and dieticians are included in the program.

### Procedures

The pilot study was conducted over a two-month period between October and November 2017. Data collection for the study was conducted over a one-year period from January to December 2018. The researcher liaised with the head of department of internal medicine at the research site to get approval and gain access to contact information of participants in the CR program. Contact information for eligible participants was provided by the records department of the hospital. The pilot study was completed with six participants to determine the practicability of the tool and outcome measure before commencing the study. The participants were contacted telephonically or by face-to-face interviews and it was found that they were able to understand and appropriately complete the questionnaire through both means of communication with the researcher. As some questions required more explanation it was found that interviews were better than self-administration of the questionnaire. No changes to the questionnaire were necessitated. The pilot study also gave the researcher an idea of the time frame required for administration of each questionnaire. It was determined that a maximum of twenty minutes was required per survey. The researcher and the researcher’s supervisor were satisfied with the results of the pilot study and proceeded to commence with the main study.

Eligible participants were contacted after the last CR session at the research site. Information sheets describing the study and consent forms were provided for those interested in participating in the study. Once verbal and written consent were obtained, the researcher administered the surveys and interviews. Face-to-face interviews took place either at the district hospital occupational therapy department or at the researcher’s private office. Telephonic interviews were conducted from the researcher’s private office. Questions that some participants chose not to answer were considered as incomplete surveys and were not included in the final sample.

### Ethics

The WHO ^14^ ethical guidelines help promote ethical conduct of research, through the enhancement and protection of the rights of the research participants and communities. The World Medical Association Helsinki Declaration ^15^ guided this study that involves human participants. Prior to participation in this study, participants gave their informed consent through a signature or verbally for telephonic surveys and interviews. Participants were informed of their right to stop participating in the study at any point, which preserved autonomy. Through safely storing audio interviews and transcription of data on a password-protected computer, confidentiality was guaranteed. Audiotaped recordings will be safeguarded for a period of two years before destroying them. Pseudonyms were used to ensure participants’ anonymity in all documentation related to the study. Counselling was made available to participants who experienced distress during participation in the study. Participation was voluntary and participants were allowed to withdraw from the study at any stage. The researcher commenced with the study only after approval from the University’s Research Ethics Committee and the Department of Health (Western Cape) was obtained.

### Validity and reliability

The Work Rehabilitation Questionnaire (WORQ) was used to ascertain the information needed to fulfil the first and second objectives. The purpose of the WORQ as an instrument is to assess work functioning in individuals participating in a vocational rehabilitation programme. After permission were obtained from the authors of the WORQ, the researchers used the WORQ in the current study. The WORQ can be administered at any point within the continuum of the RTW process and can be used with any health condition. It is aimed at gaining a fast, yet comprehensive overview of the functional problems experienced during the RTW process. The questionnaire is made up if two parts, part one being the sociodemographic and background information and part two a functional impairment section, comprising various health characteristics.

In the study conducted by Finger, Escorpizo, Bostan and De Bie, ^13^ to identify the psychometric evidence for the use of the WORQ as a valuable instrument, they concluded that the WORQ showed a high test-retest reliability and good internal consistency. Finger *et al.* ^13^ reported WORQ to have a high level of internal consistency (Cronbachs alpha=0.88) and interlinker agreement (kappa=0.82). The WORQ also exhibit acceptable levels of test retest reliability (r=0.79) and good face, content and criterion validity.

For the purpose of this study, the WORQ could be used in its English form as all participants were able to converse adequately in English.

### Analysis

The IBM SPSS software (version 25) was used to analyse, manage and generate descriptive statistics. The IBM SPSS software package is a well-established program used for batched and non-batched statistical analysis within health sciences research. Measures of central tendency, frequency and percentages for the variables describing rate of RTW were calculated. Binary logistical regression was used to explore the participant’s level of impairment and socio-demographic factors affecting RTW after the cardiac incident. The level of significance accepted in this study was p=0.05.

### Description of dependent and independent variables

The independent variables selected for this study were acquired directly for the WORQ survey. The socio-demographic variables included age, sex, civil status and level of education. Work and health-related variables from part two of the questionnaire were collated to calculate an impairment score. RTW status was captured as the dependent variable. RTW status is described as the resumption of work in the open labour market, including formal employment, informal employment and self-employment. This was captured through a yes or no answer regarding RTW after participating in a CR programme. Furthermore, the capacity of RTW included full-time work, part-time work, changes in work type or adaptation to accommodate cardiac condition.

### Univariate/descriptive statistics

To summarise sociodemographic, work and health characteristics in relation to RTW ability, descriptive statistics of central tendency, frequency and percentages were used. Summary statistics of key independent variables were also calculated.

### Bivariate analysis

**Pearson’s Chi-square test**

In order to assess the association between the independent variables (age, gender, civil status, level of education, degree of impairment) and dependent variable (RTW status) Chi-square was conducted. Significance level was set at p≤0.05.

**Binary logistical regression**

To determine the variables that influence the ability to RTW for participants with cardiac conditions, binary logistical regression was conducted. For the purpose of this study the probability of RTW after CR was projected based on participants socio-demographics, work- and health-related characteristics. The four socio-demographic variables were age, gender, civil status and level of education. The work, health and activity restriction characteristics that formed the impairment score was derived from part 2 of the WORQ, of which 37 questions from of the following ICF categories: body functions, activities and participation and environmental factors, were used. This study’s sample of n=63 could support the binary logistical regression analysis with a 95% confidence interval.

## Results

The results of the study highlights the participants’ responses to the WORQ questionnaire, which helped determine the RTW rate, factors associated with RTW and the work characteristics of the study sample.

### Summary statistics of the RTW rate

**Figure 1.**
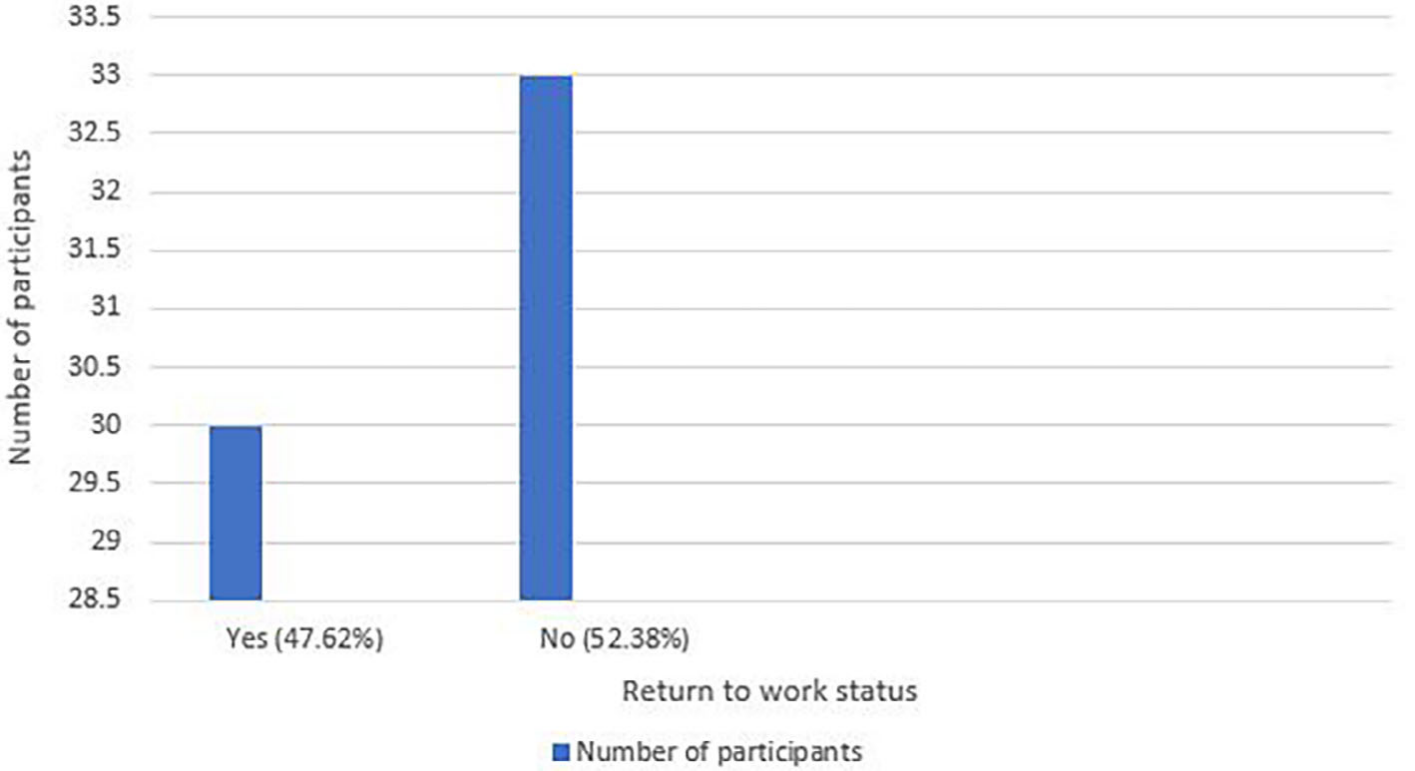
Rate of return to work.

Results of the bivariate analysis conducted to determine the RTW rate of individuals with CVD yielded the following results:

Only 30 (47.6%) of the participants reported, successful RTW after CR and 33 (52.4%) of participants did not RTW, as indicated through answering of questions four and five in the WORQ questionnaire. This was an interesting result in that despite 47.6% of individuals RTW, many of these individuals initially struggled to adapt to their worker roles immediately after completing rehabilitation ( Figure 1).

### Factors associated with return to work

Logistic regression was performed to determine the participant’s ability to RTW and each of their socio-demographic, health and work characteristics were analysed using Pearson Chi-square test, with a significant p-value that is equal to or lower than 0.05.

The model contained five independent variables. Three of the socio-demographic variables, namely civil status, education level and gender had no significant association with ability to RTW. From the logistic regression it was found that age (p≤0.004) and impairment score (p≤0.034) were significant in influencing RTW ( Tables 1 and 2).

**Table 2.** Logistic regression of socio-demographic factors and impairment score.

Variable	p-value
**Age (years)**	**0.004** *
Gender	0.088
Marital status	0.465
Education level	0.693
**Impairment score**	**0.034** *

* Statistical significance.

### Age

**Figure 2.**
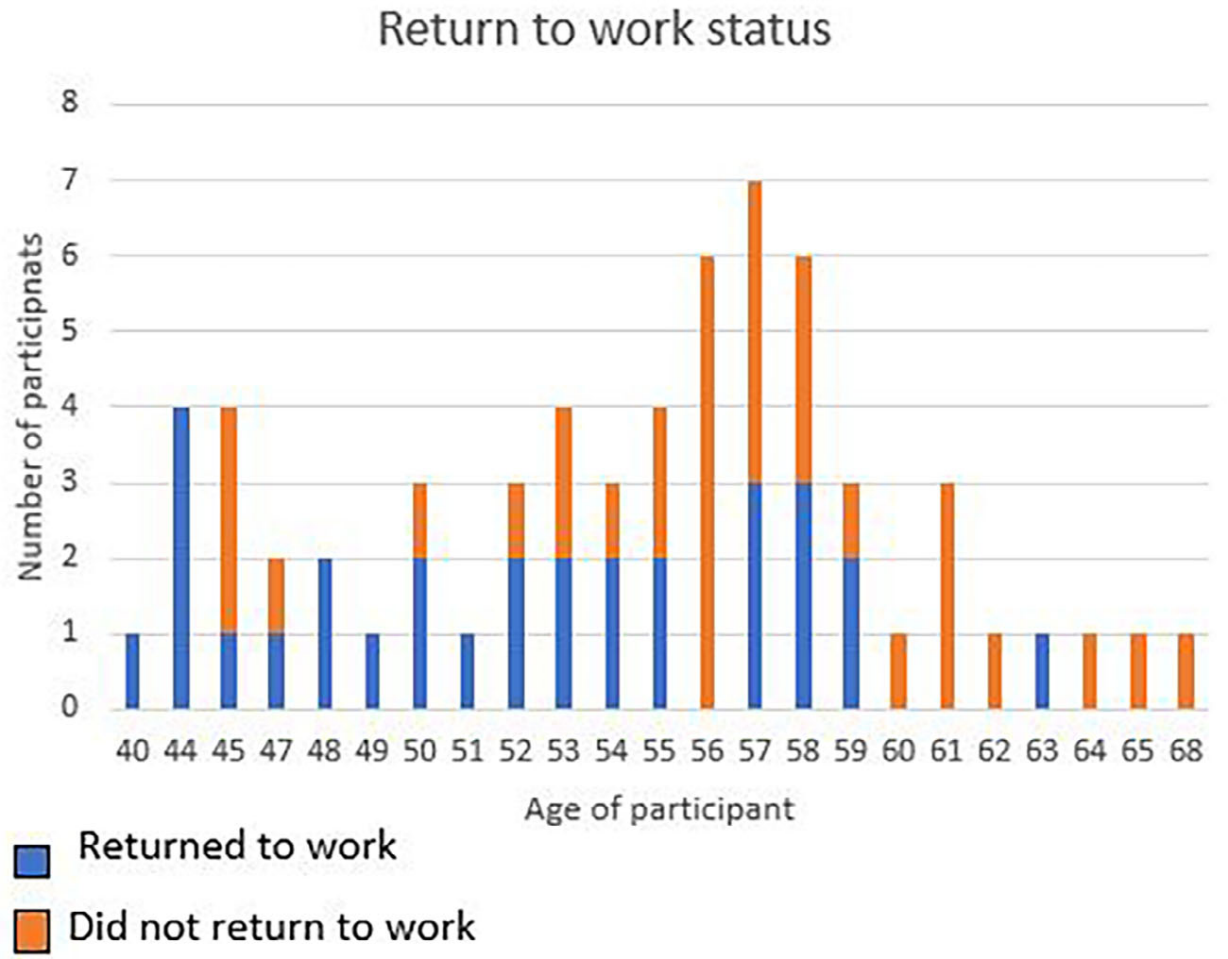
Age in relation to return to work.

From the logistic regression it was understood that the older you get the less likely you are to return to work after a cardiac incident. As noted in the figure below, most of the participants in the study sample were in the 57-year-old age group and did not RTW. All participants 44 years and below, returned to work ( Figure 2).

### Impairments and activity restrictions of study sample

A collective score from part two of the WORQ survey was calculated in order to obtain an impairment score. The survey found the impairment score to have a p-value of p=0.034, which informed the researcher that if you experience a higher degree of impairment, the less likely you are to RTW successfully ( Table 2).

**Figure 3.**
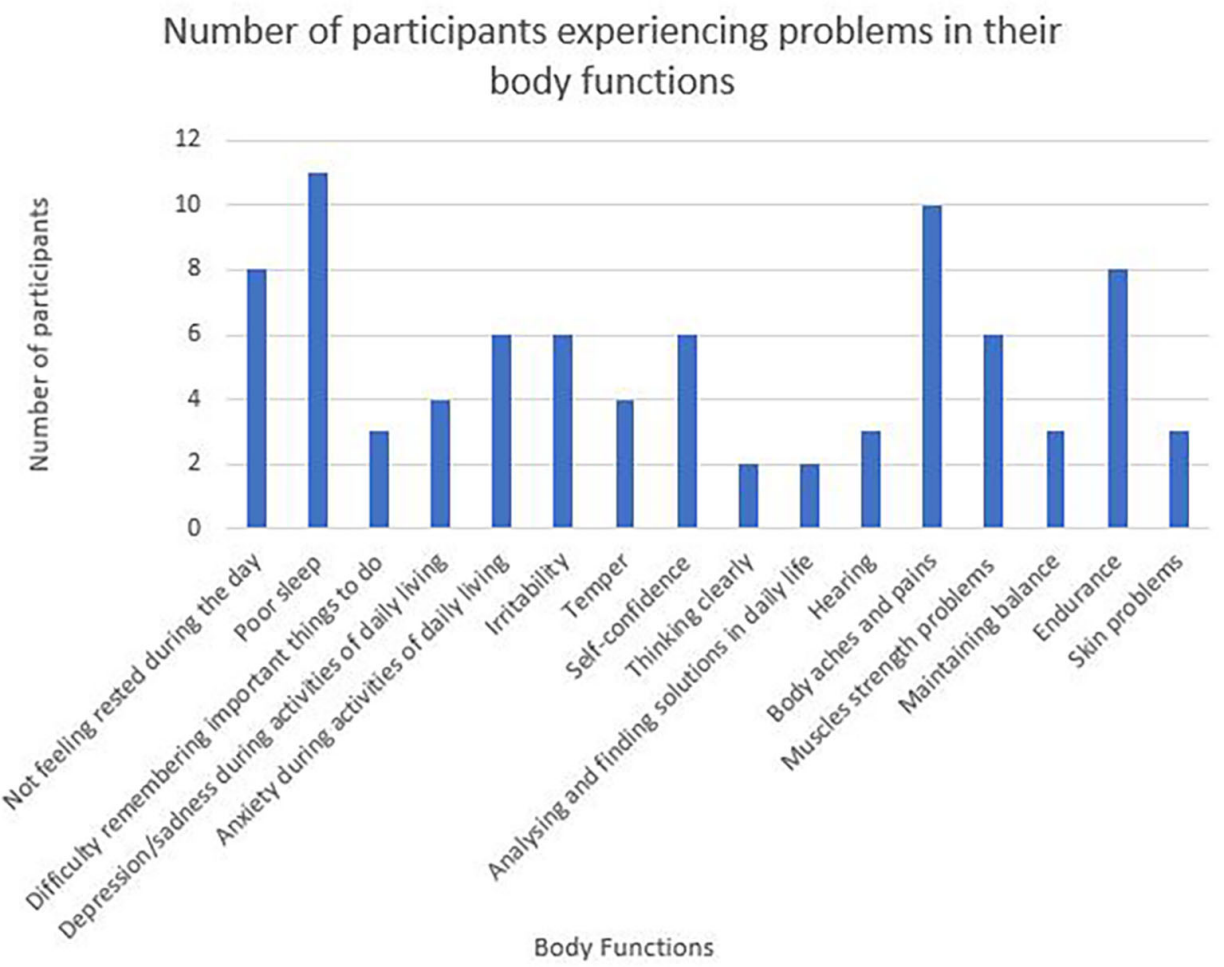
Number of participants experiencing problems in their body functions.

Sixteen categories of body function are represented in the WORQ and Figure 3 below details responses that scored 8-10 on the Likert scale for each category. Problems related to sleep, body aches and pains and endurance rated amongst the highest in the participant responses.

**Figure 4.**
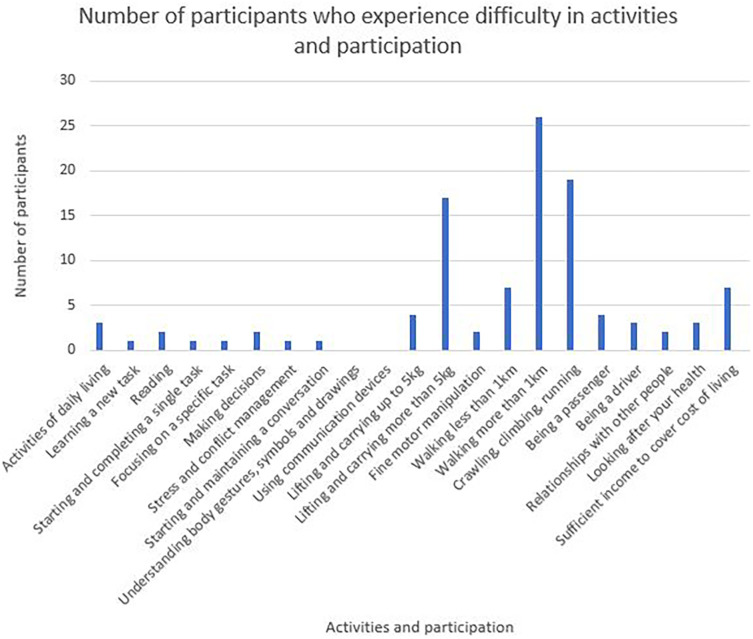
Number of participants experiencing problems in their activities and participation.

Twenty-one categories of activities and participation are represented in the WORQ and Figure 4 details responses that scored 8-10 on the Likert scale for each category. Lifting items more than 5 kg, walking more than a kilometre and crawling, climbing or running were identified as some of the most difficult activities to participate.

All of the participants found it challenging (in varying degrees) to participate in work activities, due to affected body functions or activity participation or both.

Information regarding work characteristics were also obtained from the survey. Participants were either self-employed or employed in the formal private sector or public service. Pre and post classification of work was captured. Changes were noted at most levels of classification pre and post injury, except for the one person doing heavy duty work.

## Discussion

Results of the study indicated that 52.4% of the participants did not RTW, despite having had CR ( Figure 1). Factors that significantly impacted on the participants’ ability to RTW were age and impairment score as well as work characteristics ( Table 1).

### Return to work rate after a cardiac incident

Similar to this study’s low RTW rate, in the study examining factors associated with RTW after acute myocardial infarction (AMI) in China, it was found that almost half of previously employed Chinese patients did not RTW within 12 months of the incident, with the researchers stating that CR availability was low and quality of rehabilitation poor. ^16^ However, contrary, to the Chinese and the current study, a Malaysian study that predicted RTW after a cardiac event, reported that after participating in a CR programme the RTW rate was 66.1% and that a focus on mental health during CR may improve the RTW of these individuals. ^17^ Likewise, in a Danish nationwide register follow-up study, 76.6% of patients RTW by the median time of 4 months post incidence. ^18^ The latter supports the belief that RTW after a cardiac incident can be increased successfully, taking lessons from other programs such as mental health consideration and applying it contextually.

### Socio-demographic factors that impact RTW after cardiac incident

**Age**

As reported from the quantitative results, the older you get, the less likely it is for you to RTW after a cardiac incident. Similarly, in an Italian review on the RTW after an acute cardiac incident, it was found that several studies reported that older age was an adverse factor for RTW. ^19^ Jiang et al’s. ^16^ study in China, also indicated that this notion of being older is still a negative factor for RTW. In another Danish study exploring the RTW and subsequent detachment from employment after myocardial infarction, it was also found that the 60-65 year-old age group were at the highest risk of detachment from employment. Second to this age group was the youngest age group of 30-39 years, while their analysis reported that the 40-49-year-old age group had the lowest risk of detachment from employment. ^20^ The current study is similar as all participants under the age of 44 years, RTW and participants between 60 and 65, did not RTW, excluding one 63-year-old participant. Furthermore, the Smedegaard *et al.* ^20^ study stated that within the 50-59-year-old age group, 51.7% of participants did not RTW and 48.3% of the participants did RTW. This age group’s statistics are very similar to the results of the current study with the mean age being 54 years and the rate of RTW being 47.62% and 52.38 % of participants not returning to work.

**Gender**

As reported in the results of the current study, gender did not play a significant role in determining RTW after a cardiac event. However, in the review of Fiabane *et al.* ^19^ it was found that studies reported women to be at greater risk of not returning to work and that married women, in particular, were discouraged. The studies of Kragholm *et al.* ^18^ and Jiang *et al.* ^16^ also stated that gender (i.e., males) were significantly associated with successful RTW. Within the context of this study, being located in a suburb where women are often forced to work in order to support or contribute towards the household, could be a possible reason for the insignificance.

**Marital status**

In this study 67% of the participants were married; however, marital status was not a significant factor in the RTW of this study population. Similarly, the systematic review of Cancelliere *et al.* ^21^ on factors affecting RTW after injury or illness, revealed that marital status had no association. Contrary, Dreyer *et al.* ^22^ postulates that being married, having a professional or clerical job and having no prior coronary problems were seen as more likely to RTW successfully.

**Education level**

This study also reported that educational level was not a significant factor. Contrary to these results the Italian review found that with a higher educational level and higher socio-professional category supported RTW, while blue collar workers were more at risk of not returning to work. ^19^ Similarly, the prospective cohort study on predicting RTW after Acute Mycardial Infarct found that socio-occupational factors such as self-employment, higher educational level and lower levels of depression, were predictors of RTW. ^23^ The study by Smedegaard *et al.* ^20^ on employment after MI also found that higher education was associated with successful RTW but only for men, not women. In this study, participants’ education level ranged from primary school to post-graduate level, with a majority of the study population having secondary education, however no significance was found. This could be related to the fact that blue collar work is dominant in South Africa due to education levels being lower than developed countries such as the studies mentioned above.

### Impact of impairment score on RTW after cardiac incident

This study’s calculated impairment score of p=0.034, proved to be a significant contributor to not returning to work. The higher the degree of impairment, the more likely it was for the individual to not RTW. Impaired cardiac functioning was reported to have an impact on various aspects of body functions and activities and participation which collectively affected the participants’ ability and motivation to RTW. In a study that compared young men and women returning to work after AMI, it was found that 63% of those not returning to work could be attributed to deteriorating health from impairments. ^22^ Thus, the degree of impairment must be established at the initial point of assessment and addressed within rehabilitation for it not to have such a negative influence on RTW. ^24^ In another study that compared differences between younger and older adults with multiple conditions, it was found that individuals with comorbidities were more likely to report impairments related to CVD. ^25^

### Work characteristic factors that influence RTW after cardiac incident

The type of work the individual is returning to, needs to be addressed early on in the rehabilitation process so that more individuals have the opportunity to prepare for RTW. In this study, work ranged from sedentary to heavy classifications of work. Sedentary work refers to work that involves sitting, some standing and walking with minimal lifting. Light work requires walking, standing, some pushing and pulling objects of about 5 kg. Medium work involved frequent lifting or carrying objects up to 25 kg. Lastly, heavy work is defined as frequent lifting and carrying of objects up to 50 kg. ^26^ Grace *et al.* ^27^ notes that it is imperative to discuss timing of the return to work of clients and that consideration should be given to the family’s financial situation, as well as the work characteristics ( Table 3).

**Table 3.** Work characteristics.

Variable	n (63)	%
**Pre-injury classification**		
Sedentary	14	22.2
Light	35	55.5
Medium	11	17.5
Heavy	2	3.2
Unemployed	1	1.6
**Current work classification**		
Sedentary	11	17.5
Light	24	38.1
Medium	5	7.9
Heavy	1	1.6
Unemployed	22	34.9

### Limitations to the study

The study was conducted with a small study sample, however cognisance must be taken of the fact that the district hospital mentioned in the study is the only hospital in the City of Cape Town (South Africa) that provided CR as a service to patients with cardiac conditions. There were therefore no other CR programmes available in the geographical area in the Western Cape. Therefore, due to reasons above caution must be taken in generalising the findings of the current study.

## Conclusion

The inference of these results suggests that factors associated with age and impairment and its influence on ability to RTW must be addressed more aggressively in CR programs, as well as work characteristics. The significance for an intervention program being developed is that age, degree of impairment and work characteristics can be considered when designing an integrative CR programme which includes RTW preparation and strategies.

## Data Availability

University of Western Cape: Questionnaire data for ‘Return to work rate of individuals after cardiac rehabilitation and the demographic and impairment factors that influence return to work in the Western Cape, South Africa’, https://doi.org/10.25379/uwc.21750356.v2. ^28^ Data are available under the terms of the Creative Commons Attribution 4.0 International license (CC-BY 4.0).
