# Return to work rate of individuals after cardiac rehabilitation and the demographic and impairment factors that influence return to work in the Western Cape, South Africa

**DOI:** 10.12688/f1000research.129263.3

**Published:** 2024-07-02

**Authors:** Zakeera Ganie, Shaheed Soeker, Anthea Rhoda

**Affiliations:** 1 Department of Occupational Therapy, University of the Western Cape, Bellville, Western Cape, 7535, South Africa

**Keywords:** cardiovascular disease, cardiac rehabilitation, vocational rehabilitation, return to work

## Abstract

**Background:**

Cardiovascular disease (CVD) commonly affects individuals within the working age group, often resulting in unemployment, particularly in low- to middle-income countries. The purpose of the study was to determine the return to work (RTW) rate of individuals with CVD after cardiac rehabilitation (CR) and the impact of impairment and socio-demographics on the individual’s ability to (RTW).

**Methods:**

A cross-sectional survey, namely the Work Rehabilitation Questionnaire (WORQ) was used to gather the information. The IBM SPSS software (version 25) was used to manage the statistical analysis. Individuals who completed a CR program between the ages of 18 and 64 years made up a sample of 63 research participants.

**Results:**

The RTW rate reported that only 30 (47.6%) of the participants successfully RTW after CR and 33 (52.4%) of participants did not RTW. The results also indicated that the older the individual and the higher the degree of impairment experienced, the less likely RTW would occur.

**Conclusion:**

Factors such as the age and level of functional impairment of the individual with CVD must be addressed more aggressively in CR programs, particularly if the goal of the individual with CVD is to RTW.

## Introduction

The impact that CVD in Sun-Saharan Africa has on the economy is said to be substantial as it is increasingly affecting individuals in their productive years of life. ^1^ A 10 year follow up study indicated that the incidence of Miocardial Infarct (MI) was 71.2 per 1000 males in the age group of 45-54. ^2^ Similarly in developed countries such as United States, the incidence of MI is among the working population. ^3^ Furthermore, research indicates that although the number of older individuals with MI has decreased, the number of younger individuals within the working age group, with MI has increased. ^4^ In a study conducted by Söderman, Lisspers & Sundin, ^5^ they reported that the quality of life of individuals with MI on their study decreased and that one third of these individuals did not return to work. ^6^

This burden of disease heeds calls to a more comprehensive approach for prevention and management of CVD, particularly in low- to middle-income countries such as South Africa. ^1^ Upon investigating the impact of evidence-based CR programs it was found that participation in such a preventative program does have an impact on decreasing CVD mortality and re-hospitalisation, as well as having an impact on an individual’s ability to RTW. ^2^ A prospective study conducted with 83 patients of working age during their CR found that job satisfaction and a positively perceived work environment resulted in early RTW after cardiac intervention and may have economic benefits and improve quality of life. ^3^ In a meta-analysis conducted by Sadeghi *et al.*, ^7^ they indicate the positive affect of cardiac rehabilitation programs in facilitating the return to work of individuals diagnosed with cardiovascular conditions. The above authors indicate that of the results of the 16 studies analysed, pooled results showed that the prevalence of RTW in patients attending the cardiac rehabilitation (CR) group was 66% and the control group was 58%. They further indicate that subgroup analysis reveals that the proportion of RTW was higher in white-collar classified jobs, i.e. 76%, compared to individuals who had blue-collar classified jobs, i.e. 63%. A further analysis revealed that the RTW rate for individuals who attended out-patient CR was 72% and was more effective when compared to individuals that attended in-patient CR, the RTW rate being 62%.

Slebus *et al.* ^8^ further explored facilitating factors for RTW and identified the following: no signs and symptoms of the disease, work contentment, positive relationships at work, ability to participate in work activities, information from the doctor that encouraged RTW, medical care was working well, family relationships were positive, financial motivation and intrinsic motivation to work. Similar results from a qualitative study of perpetuating factors for long-term sick leave and promoting factors for RTW, found that illness perceptions and self-efficacy expectations can be promoting factors for RTW. ^9^ Furthermore, financial obligations and culture are also contributing factors of RTW according to a study that reviewed the employment status after myocardial infarction (MI) among men. These men stated that they did not want to take advantage of their wives earning an income and that it was their responsibility as the breadwinner to provide for their families. ^10^

In a Danish cohort study examining RTW after hospitalisation for heart failure, the authors speculate that a lack of RTW was due to functional limitations as a result of the disease as well as the psychological effects of having the heart failure diagnosis. ^11^ Kearney *et al.* ^12^ also observed that fatigue was most frequently reported as a barrier to RTW, followed by mild cognitive impairment such as impaired memory and cognitive processing. Similarly, a study with patients who had implantable cardioverter defibrillators estimated that 75% of patients experience mild to severe short-term cognitive impairment, with roughly 33% of these expected to be prolonged at six months. ^13^

Patients suffering from anxiety or emotional distress following the cardiac incident have been found to have increased difficulty in lifestyle modification and less likely to complete CR. ^14^ This could have a negative impact on the individual’s prospects of RTW and successfully maintaining employment. Similarly, in a prospective cohort study examining the associations between depressive episodes and anxiety disorder with RTW after MI at three and six months found that the presence of a depressive episode and anxiety disorder during the first three months after the MI was a significant predictor of not RTW by 12 months. ^15^

Kearney *et al.* ^12^ also found that patients in occupations such as labourers, tradesman and in the transport industry were less likely to RTW as their jobs were labour-intensive and that their illness had a greater impact on their ability to perform their work tasks.

The significance of the current study was to determine the demographic factors that are associated with RTW for individuals with cardiac conditions. The results would determine the RTW rate of individuals after they have completed a CR program. Furthermore, the study would determine which specific factors influence RTW for individuals with cardiac conditions after the completion of a CR program.

### Aim and objectives

• To determine the RTW rate of individuals with CVD after a completing a CR program
• To determine the impact of impairment and socio-demographic factors on an individual’s ability to RTW after completing a CR program

## Methods

### Participant recruitment

All participants were patients of a district hospital, who were admitted due to a cardiac incident between 2017 and 2018. Each participant was referred to CR as an outpatient. The sampling technique utilised in this study was convenience sampling. A convenient sample was utilised at the data collection site. The advantage of using this sampling technique is that it is inexpensive, and results can be sorted quickly. However, there is a risk of the sample not being representative. ^16^

#### Inclusion criteria

The inclusion criteria for participation was an age of 18-64 years, completion of the CR program and active employment prior to the cardiac incident.

### Procedures

A pilot study was conducted over a two-month period between October and November 2017. Data collection for the study was conducted over a one-year period from January to December 2018. After ethical approval, access to contact information of participants in the CR program was obtained from the hospitals records department. The pilot study was completed with six participants to determine the practicability of the tool and outcome measure before commencing the study. Some participants required assistance with reading or comprehension therefore it was found that interviews were better than self-administration of the questionnaire. No changes to the questionnaire were necessitated. The questions were read verbatim. Through the pilot study it was determined that a maximum of twenty minutes was required per survey.

Information sheets describing the study and consent forms were provided for those interested in participating in the study. Once verbal and written consent were obtained, surveys were administered. Questions that some participants chose not to answer were considered as incomplete surveys and were not included in the final sample. Research participants chose to not answer questions that did not related to their context and life circumstances, e.g. they did not focus on commenting on their type of work they would want to do in the future as they were unemployed. In terms of the analysis only completed questionnaires were used.

### Ethics

The World Health Organisation’s (WHO) ^17^ ethical guidelines helped promote the ethical conduct of research. Participants were informed of their right to stop participating in the study at any time during the study. Counselling was made available to participants who experienced distress during participation in the study. Through safely storing audio interviews and transcription of data on a password-protected computer, confidentiality was guaranteed. Pseudonyms were used to ensure participants’ anonymity in all documentation related to the study. The study commenced after approval from the University’s Research Ethics Committee and the Department of Health (Western Cape) was obtained.

### Validity and reliability

The WORQ is aimed at gaining a fast, yet comprehensive overview of the functional problems experienced during the RTW process. The questionnaire is made up if two parts, part one being the sociodemographic and background information and part two a functional impairment section, comprising various health characteristics.

A study reported that the WORQ has a high level of internal consistency (Cronbachs alpha=0.88) and interlinker agreement (kappa=0.82), exhibits acceptable levels of test retest reliability (r=0.79), good face, content and criterion validity, therefore a valuable and appropriate instrument to use. ^18^

### Analysis

The independent variables selected for this study were acquired directly for the WORQ survey. The socio-demographic factors that made up the independent variables included age, sex, civil status and level of education. Work and health-related variables from part two of the questionnaire were collated to calculate an impairment score. RTW status was captured as the dependent variable. RTW status in the current study was described as the resumption of work in the open labour market, including formal employment, informal employment and self-employment. This was captured through a yes or no answer regarding RTW after participating in a CR programme. Furthermore, the capacity of RTW included full-time work, part-time work, changes in work type or adaptation to accommodate cardiac condition.

To summarise sociodemographic, work and health characteristics in relation to RTW ability, descriptive statistics of central tendency, frequency and percentages were used. Summary statistics of key independent variables were also calculated.

In order to assess the association between the independent variables (age, gender, civil status, level of education, degree of impairment) and dependent variable (RTW status) Chi-square was conducted. Significance level was set at p≤0.05.

To determine the variables that influence the ability to RTW for participants with cardiac conditions, binary logistical regression was conducted. For the purpose of this study the probability of RTW after CR was projected based on participants socio-demographics, work- and health-related characteristics. The work, health and activity restriction characteristics that formed the impairment score was derived from part 2 of the WORQ, of which 37 questions from of the following ICF categories: body functions, activities and participation and environmental factors, were used. This study’s sample of n=63 supported the binary logistical regression analysis with a 95% confidence interval.

## Results

The results of the study highlights the participants’ responses to the WORQ questionnaire, which helped determine the RTW rate, factors associated with RTW and the work characteristics of the study sample. Seventy-seven copies of the questionnaire were administered, however only 63 surveys were accepted for analysis. Fourteen surveys were incomplete and therefore voided. The time period after completing of the CR varied amongst participants.

The study sample comprised 27 female and 36 male participants, with a mean age of 54 years and a standard deviation of 5.95. Most (60.3%) of the participants in the sample were in the 51-60 year age group. Forty-two of the participants were married, 12 divorced, 7 never married, 1 widowed and 1 was cohabiting. The majority (73%) of participants had a secondary school education. Forty-two participants completed secondary school, with 8 graduates from college/university, 2 had completed post-graduate studies and 7 primary school only. All the participants had experienced a MI between 2016 and 2018 and received medical and therapeutic intervention. Nineteen participants were receiving government support in the form of social grants ( Table 1).

**Table 1.** Logistic regression of socio-demographic factors and impairment score.

Variable	Odds ratio	Confidence interval-lower	Confidence interval-upper	P-value
Age	9.280	1.085	79.395	0.004 *
Gender	2.125	.766	5.898	0.088
Marital status	.563	.195	1.621	0.456
Education	3.111	.577	16.783	0.693
Impairment score	.500	.388	.644	0.034 *

* Statistical significance.

### Summary statistics of the RTW rate

**Figure 1.**
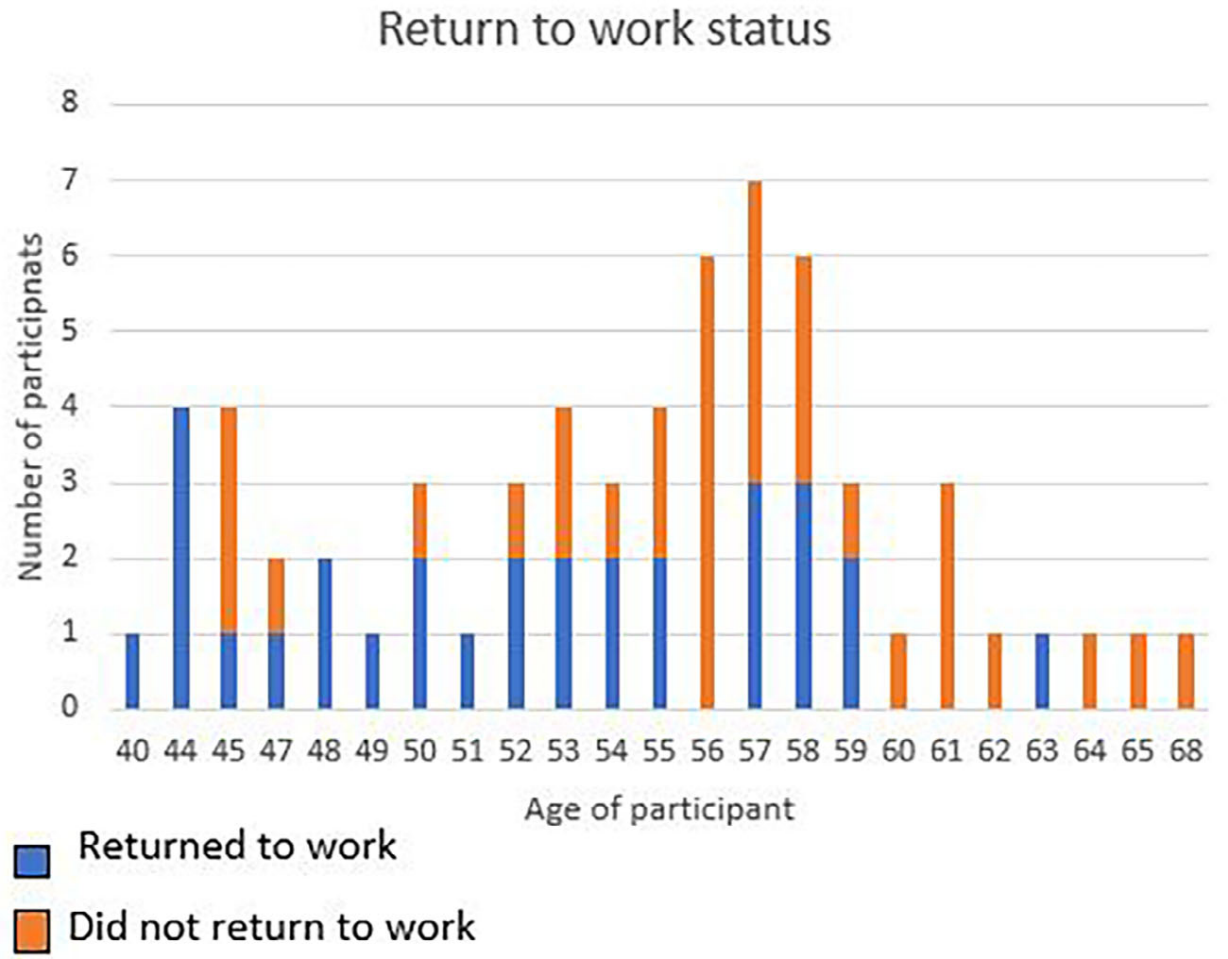
Age in relation to return to work.

Results of the bivariate analysis conducted to determine the RTW rate of individuals with CVD yielded the following results:

Only 30 (47.6%) of the participants reported, successful RTW after CR and 33 (52.4%) of participants did not RTW. ( Figure 1).

### Factors associated with RTW

There were five independent variables. Three of the socio-demographic variables, namely civil status, education level and gender had no significant association with ability to RTW. From the logistic regression it was found that age (p≤0.004) and impairment score (p≤0.034) were significant in influencing RTW ( Table 1).

### Age

From the logistic regression it was understood that the older you get the less likely you are to RTW after a cardiac incident. As noted in the figure below, most of the participants in the study sample were in the 57-year-old age group and did not RTW. All participants 44 years and below, did RTW ( Figure 1).

### Impairments and activity restrictions of study sample

The survey found the impairment score to have a p-value of p=0.034, indicating that if you experience a higher degree of impairment, the less likely you are to RTW successfully. Responses that scored 8-10 on the Likert scale for each category are detailed below.

**Figure 2.**
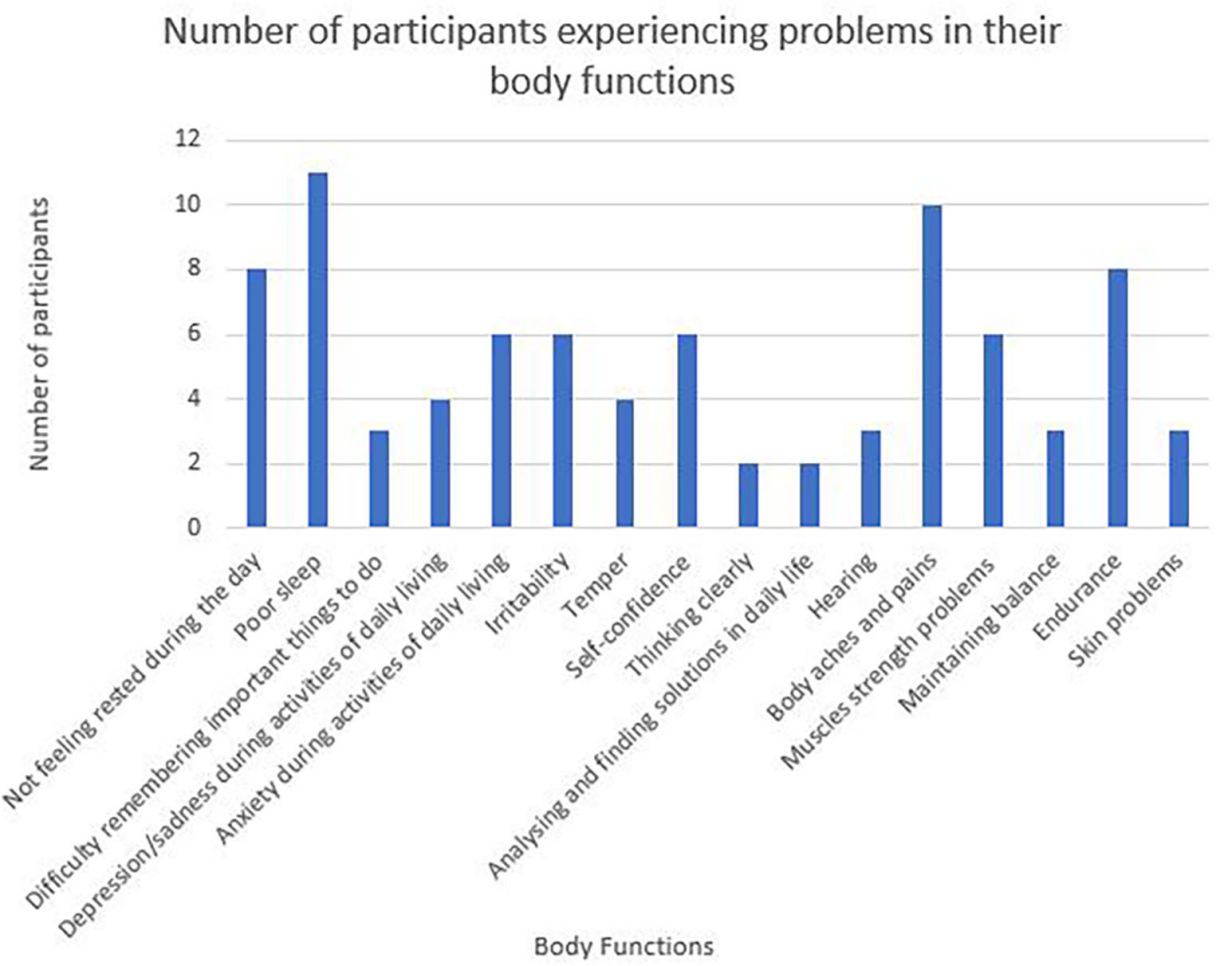
Number of participants experiencing problems in their body functions.

Sixteen categories of body function are represented in the WORQ ( Figure 2). Problems related to sleep, body aches and pains and endurance rated amongst the highest in the participant responses.

**Figure 3.**
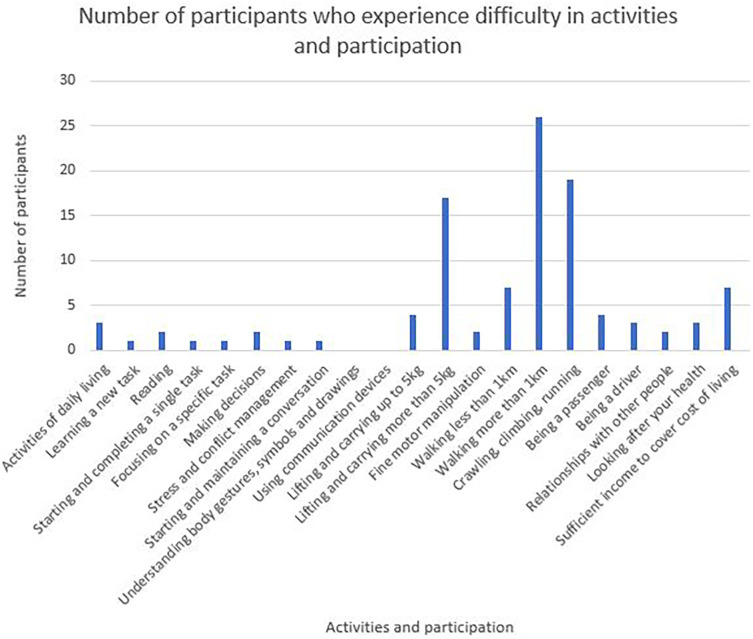
Number of participants experiencing problems in their activities and participation.

Twenty-one categories of activities and participation are represented in the WORQ ( Figure 3). Lifting items more than 5 kg, walking more than a kilometre, crawling, climbing or running were identified as some of the most difficult activities to participate in.

Changes were noted at most levels of classification pre and post injury, except for the one person doing heavy duty work. ( Table 2).

**Table 2.** Work characteristics.

Variable	n (63)	%
**Pre-injury classification**		
Sedentary	14	22.2
Light	35	55.5
Medium	11	17.5
Heavy	2	3.2
Unemployed	1	1.6
**Current work classification**		
Sedentary	11	17.5
Light	24	38.1
Medium	5	7.9
Heavy	1	1.6
Unemployed	22	34.9

## Discussion

Results of the study indicated that 52.4% of the participants did not RTW, despite having had CR ( Figure 1). Factors that significantly impacted on the participants’ ability to RTW were age and degree of impairment as well as work characteristics ( Table 2).

### RTW rate after a cardiac incident

Similar to this study’s low RTW rate, in the study examining factors associated with RTW after acute myocardial infarction (AMI) in China, it was found that almost half of the previously employed Chinese patients did not RTW within 12 months of the incident, with the researchers stating that CR availability was low and quality of rehabilitation poor. ^19^ However, contrary, to the Chinese and the current study, a Malaysian study that predicted RTW after a cardiac event, reported that after participating in a CR program the RTW rate was 66.1% and that a focus on mental health during CR may improve the RTW of these individuals. ^20^ Likewise, in a Danish nationwide register follow-up study, 76.6% of patients RTW by the median time of 4 months post incidence. ^21^ The latter supports the belief that RTW after a cardiac incident can be increased successfully, taking lessons from other programs such as mental health into consideration and applying it contextually.

### Socio-demographic factors that impact RTW after cardiac incident

As reported from the quantitative results, the older you get, the less likely it is for you to RTW after a cardiac incident. Similarly, in an Italian review on the RTW after an acute cardiac incident, it was found that several studies reported that older age was an adverse factor for RTW. ^22^ Jiang *et al’s*. ^19^ study in China, also indicated that this notion of being older is still a negative factor for RTW. In another Danish study exploring the RTW and subsequent detachment from employment after MI, it was also found that the 60-65 year-old age group were at the highest risk of detachment from employment. Second to this age group was the youngest age group of 30-39 years, while their analysis reported that the 40-49-year-old age group had the lowest risk of detachment from employment. ^23^ The current study is similar as all participants under the age of 44 years, RTW and participants between 60 and 65, did not RTW, excluding one 63-year-old participant. Furthermore, the Smedegaard *et al.* ^23^ study stated that within the 50-59-year-old age group, 51.7% of participants did not RTW and 48.3% of the participants did RTW. These statistics are very similar to the results of the current study with the mean age being 54 years and the rate of RTW being 47.62% and 52.38 % of participants not RTW.

As reported in the results of the current study, gender did not play a significant role in determining RTW after a cardiac event. However, in the review of Fiabane *et al.* ^22^ it was found that studies reported women to be at greater risk of not RTW and that married women, in particular, were discouraged. The studies of Kragholm *et al.* ^21^ and Jiang *et al.* ^19^ also stated that gender (i.e., males) were significantly associated with successful RTW. Within the context of this study, being located in a suburb where women are often forced to work in order to support or contribute towards the household, could be a possible reason for the insignificance.

In this study 67% of the participants were married; however, marital status was not a significant factor in the RTW of this study population. Similarly, the systematic review of Cancelliere *et al.* ^24^ on factors affecting RTW after injury or illness, revealed that marital status had no association. Contrary, Dreyer *et al.* ^25^ postulates that being married, was seen as more likely to RTW successfully.

The current study also reported that educational level was not a significant factor. Contrary to these results the Italian review found that with a higher educational level and higher socio-professional category supported RTW, while blue collar workers were more at risk of not RTW. ^22^ Similarly, the prospective cohort study on predicting RTW after AMI found that socio-occupational factors such as self-employment, higher educational level and lower levels of depression, were predictors of RTW. ^26^ The study by Smedegaard *et al.* ^23^ on employment after MI also found that higher education was associated with successful RTW but only for men, not women. In this study, participants’ education level ranged from primary school to post-graduate level, with a majority of the study population having secondary education, however no significance was found. This could be related to the fact that blue collar work is dominant in South Africa due to education levels being lower than developed countries such as the studies mentioned above.

### Impact of impairment score on RTW after cardiac incident

This study’s calculated impairment score of p=0.034, proved to be a significant contributor to not RTW. The higher the degree of impairment, the more likely it was for the individual to not RTW. Impaired cardiac functioning was reported to have an impact on various aspects of body functions and activities and participation which collectively affected the participants’ ability and motivation to RTW. In a study that compared young men and women RTW after AMI, it was found that 63% of those not RTW could be attributed to deteriorating health from impairments. ^25^ Thus, the degree of impairment must be established at the initial point of assessment and addressed within rehabilitation for it not to have such a negative influence on RTW. ^27^ In another study that compared differences between younger and older adults with multiple conditions, it was found that individuals with comorbidities were more likely to report impairments related to CVD. ^28^

### Work characteristic factors that influence RTW after cardiac incident

The type of work the individual is returning to, needs to be addressed early on in the rehabilitation process so that more individuals have the opportunity to prepare for RTW. In this study, work ranged from sedentary to heavy classifications of work. Sedentary work refers to work that involves sitting, some standing and walking with minimal lifting. Light work requires walking, standing, some pushing and pulling objects of about 5 kg. Medium work involved frequent lifting or carrying objects up to 25 kg. Lastly, heavy work is defined as frequent lifting and carrying of objects up to 50 kg. ^29^ Grace *et al.* ^30^ notes that it is imperative to discuss timing of the RTW of clients and that consideration should be given to the family’s financial situation, as well as the work characteristics. Considerations regarding the individuals mental health and modifiable tasks should be done in partnership with the individuals employer. ^31^

### Limitations to the study

The study was conducted with a small study sample, however cognisance must be taken of the fact that the district hospital mentioned in the study is the only hospital in the City of Cape Town (South Africa) that provided CR as a service to patients with cardiac conditions. There were no other CR programs available in the geographical area in the Western Cape. Due to reasons above caution must be taken in generalising the findings of the current study.

## Conclusion

The aim and objectives of the study were to determine the RTW rate of individuals with CVD after a cardiac incident and the impact of impairment and socio-demographic factors on an individual’s ability to RTW. The results of this study suggests that factors associated with age and impairment influences one’s ability to RTW. Work characteristics changes were evident upon RTW after the cardiac incident. Civil status, education level and gender had no significance to RTW. It is therefore imperative for intervention programs being developed to consider age, degree of impairment and work characteristics when designing an integrative CR program which includes RTW preparation and strategies.

## Data availability

### Underlying data

University of Western Cape: Questionnaire data for ‘Return to work rate of individuals after cardiac rehabilitation and the demographic and impairment factors that influence return to work in the Western Cape, South Africa’, https://doi.org/10.25379/uwc.21750356.v2. ^32^

Data are available under the terms of the Creative Commons Attribution 4.0 International license (CC-BY 4.0).
