# Supplementary material for: Return to work rate of individuals after cardiac rehabilitation and the demographic and impairment factors that influence return to work in the Western Cape, South Africa
Source: F1000Res. 2024 Jul 2;12:894. Originally published 2023 Jul 27. [Version 3] doi: 10.12688/f1000research.129263.3 (PMC11016172; doi:10.12688/f1000research.129263.3)
Supplement: Peer review history

# Reviewer report: Reviewer response for version 3

**DOI:** 10.5256/f1000research.168376.r298228

**Published:** 2024-07-15

**Authors:** Vrati M. Mehra

**Affiliations:** 1 Temerty Faculty of Medicine, University of Toronto, Toronto, Ontario, Canada

I think the paper looks much improved. I believe it is ready for indexed.

Is the work clearly and accurately presented and does it cite the current literature?

Partly

If applicable, is the statistical analysis and its interpretation appropriate?

Partly

Are all the source data underlying the results available to ensure full reproducibility?

Partly

Is the study design appropriate and is the work technically sound?

Partly

Are the conclusions drawn adequately supported by the results?

Yes

Are sufficient details of methods and analysis provided to allow replication by others?

Partly

Reviewer Expertise:

NA

I confirm that I have read this submission and believe that I have an appropriate level of expertise to confirm that it is of an acceptable scientific standard.

# Reviewer report: Reviewer response for version 2

**DOI:** 10.5256/f1000research.158815.r262503

**Published:** 2024-04-13

**Authors:** Vrati M. Mehra

**Affiliations:** 1 Temerty Faculty of Medicine, University of Toronto, Toronto, Ontario, Canada

Introduction:

 The statement: "In most countries the incidence of MI is among the working population." references a study done on working age population in the United States alone. As such it is not the best study to show that MI is increasing globally and specifically in the working age population. A good source maybe the recent report from Global Burden of Cardiovascular Diseases. 

 Similarly please reconsider the reference for the statement "Furthermore, research indicates that although the number of older individuals with MI has decreased, the number of younger individuals with MI has increased" as the current study cited does not state that MIs have increased in younger populations and decreased in older populations. 

 Kindly add "after completing a CR program" to the Aims and objectives so that they accurately describe that you are studying RTW only among those who completed a CR program. 

 "To determine the RTW rate of individuals with CVD after a cardiac incident after completing a CR program."

 "To determine the impact of impairment and socio-demographic factors on an individual’s ability to RTW after a cardiac incident after completing a CR program.

 Methods

 Procedures

 Kindly specify what were some reasons for people leaving questions incomplete. 

 I would suggest including a separate section in the Methods section that defines how each of the independent variables was coded and which option was treated as the reference option within each of these. 

 Methods, Analysis: Kindly specify how many months (SD) after the completion of CR were the participants interviewed about returning to work. 

 Results

 The results mentioned in Paragraph 2 should be included in a table. The current Table 1 only includes p values for each variable. 

 Table 1 should be modified to include categories where applicable for each of the variables. The Odds ratios, CI and P value. This is usually the way logistic regression results are reported in the literature. 

 Discussion, Work characteristic factors that influence RTW after cardiac incident:

 I would suggest that the authors also consider the importance of modification of duties at work after CVD so people can continue to work if they would like to do so. As well helping patients learn ways to reintegrate in work safely is also important.

Is the work clearly and accurately presented and does it cite the current literature?

Partly

If applicable, is the statistical analysis and its interpretation appropriate?

Partly

Are all the source data underlying the results available to ensure full reproducibility?

Partly

Is the study design appropriate and is the work technically sound?

Partly

Are the conclusions drawn adequately supported by the results?

Yes

Are sufficient details of methods and analysis provided to allow replication by others?

Partly

Reviewer Expertise:

Epidemiology, Logistic Regression, Large survey dataset analysis.

I confirm that I have read this submission and believe that I have an appropriate level of expertise to confirm that it is of an acceptable scientific standard, however I have significant reservations, as outlined above.

# Response

**Published:** 2024-06-24

**Authors:** Shaheed Soeker

**Affiliations:** University of the Western Cape, South Africa

Introduction:

 1) The statement: "In most countries the incidence of MI is among the working population." references a study done on working age population in the United States alone. As such it is not the best study to show that MI is increasing globally and specifically in the working age population. A good source maybe the recent report from Global Burden of Cardiovascular Diseases. 

 2) The first reference is in Sub-Saharan Africa.

 Change sentence to indicate developed countries such as the United States experience the same thing.

 3)Similarly please reconsider the reference for the statement "Furthermore, research indicates that although the number of older individuals with MI has decreased, the number of younger individuals with MI has increased" as the current study cited does not state that MIs have increased in younger populations and decreased in older populations. 

 The intention was to illustrate that the working population is now increasingly affected by MI.

 Restructured the sentence to indicate that.

 4) Kindly add "after completing a CR program" to the Aims and objectives so that they accurately describe that you are studying RTW only among those who completed a CR program. 

 "To determine the RTW rate of individuals with CVD after a cardiac incident after completing a CR program."

 "To determine the impact of impairment and socio-demographic factors on an individual’s ability to RTW after a cardiac incident after completing a CR program.

 5) Added ‘after completing CR program …’ to aims and objectives.

 6) Procedures

 Kindly specify what were some reasons for people leaving questions incomplete. 

 7) Personal choice.

 Research participants chose to not answer questions that did not related to their context and life circumstances, e.g. they did not focus on commenting on their type of work they would want to do in the future as they were unemployed. In terms of the analysis only completed questionnaires were used.      

 8) I would suggest including a separate section in the Methods section that defines how each of the independent variables was coded and which option was treated as the reference option within each of these. 

 Independent variables: Education, work, age, gender, marital status, medical condition. Sentence rephrased to indicate the socio-demographic made up the independent variables. 

 Dependent variable: Return work rate   

 9) Methods, Analysis: Kindly specify how many months (SD) after the completion of CR were the participants interviewed about returning to work. 

 10) Varied but a min of 1 month  as the cardiac rehab programme needed to be complete first.

 Added this to the results section.

 11) The results mentioned in Paragraph 2 should be included in a table. The current Table 1 only includes p values for each variable. 

 12) Added a separate table with demographic details be included. See below Table 1.

 Table 1 should be modified to include categories where applicable for each of the variables. The Odds ratios, CI and P value. This is usually the way logistic regression results are reported in the literature. 

 13) Table has been modified to include the Odds ratio and CI.

 14) Discussion, Work characteristic factors that influence RTW after cardiac incident:

 I would suggest that the authors also consider the importance of modification of duties at work after CVD so people can continue to work if they would like to do so. As well helping patients learn ways to reintegrate in work safely is also important.

 15) Considerations regarding the individuals mental health and  modifiable tasks should be done in partnership with the individuals employer.

 Ref:

 Predicting return to work following myocardial infarction: A Prospective Longitudinal Cohort Study

 Sun, W., Gholizadeh, l., Perry, l., Kang, K

Int J Environ Res Public Health.

 19(13)

 2022

# Reviewer report: Reviewer response for version 2

**DOI:** 10.5256/f1000research.158815.r224043

**Published:** 2024-01-24

**Authors:** Thomas Maribo

**Affiliations:** 1 Public Health, Aarhus University, Aarhus C, Denmark

Thank you for the revised version of the manuscript. It has improved the article. I have no further comments.

Is the work clearly and accurately presented and does it cite the current literature?

No

If applicable, is the statistical analysis and its interpretation appropriate?

Partly

Are all the source data underlying the results available to ensure full reproducibility?

Partly

Is the study design appropriate and is the work technically sound?

Yes

Are the conclusions drawn adequately supported by the results?

No

Are sufficient details of methods and analysis provided to allow replication by others?

No

Reviewer Expertise:

Cardiac rehabiltiation. vocational rehabilitation

I confirm that I have read this submission and believe that I have an appropriate level of expertise to confirm that it is of an acceptable scientific standard.

# Reviewer report: Reviewer response for version 1

**DOI:** 10.5256/f1000research.141934.r200453

**Published:** 2023-10-18

**Authors:** Barbara Iansã de Lima Barroso

**Affiliations:** 1 Federal University of São Paulo, São Paulo, Brazil

The article deals with cardiovascular diseases and the return to work of people after cardiac rehabilitation (CR) in low-to-middle income countries.

 The article is of great importance for the Return to Work area, however, some adjustments need to be made. Getting started:

1. Review the references used, bring more current scientific evidence, systematic reviews and population studies are well evaluated for a more robust introduction and discussion;
2. The word return-to-work must be written without a hyphen, although an Entry Terms version with a hyphen appears in MeSH, in current literature we use the word without a hyphen.
3. Abbreviations throughout the text need to be revised, provide only one complete sentence followed by the abbreviation, then present only the abbreviation.
4. In statistical analysis, authors need to scientifically support the choice of method, and not just sampling (this was done to support the convenience sample), please provide more details;
5. Results can start with a description of the sample; 
6. The conclusion needs to be redone. Add whether the objectives of the study were achieved, the limits of the study, the strengths and divergent points of the research.

I hope my contributions can further improve your work. Everything about RTW is very challenging, don't give up, continue to walk your path talking about work and its importance in our lives.

Is the work clearly and accurately presented and does it cite the current literature?

Partly

If applicable, is the statistical analysis and its interpretation appropriate?

Partly

Are all the source data underlying the results available to ensure full reproducibility?

Yes

Is the study design appropriate and is the work technically sound?

Yes

Are the conclusions drawn adequately supported by the results?

No

Are sufficient details of methods and analysis provided to allow replication by others?

Yes

Reviewer Expertise:

I work mainly in the field of Policy, Planning and Management of the Brazilian Unified Health System, and its policies for the LGBTQIAP+ population. I have experience and interest in the area of Public Health, in the following topics: male and female workers' health - contributions on health, work and society; precariousness of work; health work; management in Occupational Health, and Systematic Reviews in the field of Work.

I confirm that I have read this submission and believe that I have an appropriate level of expertise to confirm that it is of an acceptable scientific standard, however I have significant reservations, as outlined above.

# Response

**Published:** 2023-11-10

**Authors:** Shaheed Soeker

**Affiliations:** University of the Western Cape, South Africa

**Response to reviewers**

 Dear Professor

 Thank you so much for the informed comments that you provided.

 I have provided a detailed response to the comments that you have provided. As a team we have tried to incorporate as many of the suggestions that you provided.

 Reviewer 1: **Barbara Iansã de Lima Barroso**, Federal University of São Paulo, São Paulo, Brazil 

**Comment**

 Review the references used, bring more current scientific evidence, systematic reviews and population studies are well evaluated for a more robust introduction and discussion;

**Response**

 The introduction and discussion was updated with scientific evidence from systematic reviews

**Comment**

 The word return-to-work must be written without a hyphen, although an Entry Terms version with a hyphen appears in MeSH, in current literature we use the word without a hyphen.

**Response**

 Hyphen has been removed.

**Comment**

 Abbreviations throughout the text need to be revised, provide only one complete sentence followed by the abbreviation, then present only the abbreviation.

**Response**

 Abbreviations have been revised.

**Comment**

 In statistical analysis, authors need to scientifically support the choice of method, and not just sampling (this was done to support the convenience sample), please provide more details;

 Response

 Reference has been added:

 Kaur P, Stolzfus J, Yellapu V: Descriptive statistics. *Int j Acad Med. *2018; 4: 60-63

**Comment**

 Results can start with a description of the sample; 

**Response**

 Sample description has been moved from methods to results section.

**Comment**

 The conclusion needs to be redone. Add whether the objectives of the study were achieved, the limits of the study, the strengths and divergent points of the research.

**Response**

 Conclusion has been rewritten.

# Reviewer report: Reviewer response for version 1

**DOI:** 10.5256/f1000research.141934.r206156

**Published:** 2023-09-26

**Authors:** Thomas Maribo

**Affiliations:** 1 Public Health, Aarhus University, Aarhus C, Denmark

Headings should align with a scientific paper and headings like “description of dependent and independent variable”, “Univariate/descriptive statistics”, and “bivariate analysis” should be removed.

 Procedure starts with "The pilot study" – which pilot study? If I understand correct you could start with "A pilot study".

 In the methods section: place the statistical programme at the end, not the beginning.

 Delete redundant sentences like “The IBM SPSS software package is a well-established program used for batched and non-batched statistical analysis within health sciences research.” (this is a scientific paper not a text book).

 In the methods section, the impairment score is mentioned in 2 or 3 places. Rewrite and describe the outcome measures and then the analyses.

 The term “the researcher” is used far too much (8 times!). The scientific world does not care what and how the researcher did - it is the study that is important. Rephrase “The researcher liaised with the head of department of internal medicine” - and more like that. And remove "The researcher and the researcher’s supervisor were satisfied" we do not care what your supervisor think or do. We want science!

 Rewrite and shorten "Ethics". This is far too long with redundant sentences like "The WHO14 ethical guidelines help promote ethical conduct of research, through the enhancement and protection of the rights of the research participants and communities" – this is a scientific paper! "Participation was voluntary and participants were allowed to withdraw from the study at any stage." is redundant as is it already stated.

 The results section should start with a description of the population: how many patients were enrolled in CR, how many completed, how many (and who) did not complete CR – and how many did not complete the questionnaire

 The sentence "Logistic regression was performed to determine the participant’s ability to RTW and each of their socio-demographic, health and work characteristics were analysed using Pearson Chi-square test, with a significant p-value that is equal to or lower than 0.05." is placed in the results section! move to the methods section. If you want other researchers to read your paper it must be rewritten. I urge you to consult an experienced scientist.

 Rewrite the discussion and include the following:

- why did you use interview and not the questionnaire? What did that mean?
- Why were interviews held at the first authors private place? And could this bias the result?

Minor:

 Reread and check for abbreviations. cardiac rehabilitation (CR) is abbreviated in the 1st paragraph, yet cardiac rehabilitation is written out 3 times.

 Rewrite " In the study conducted by Finger, Escorpizo, Bostan and De Bie,13 to identify the psychometric evidence for the use of the WORQ as a valuable instrument, they concluded that the WORQ showed a high test-retest reliability and good internal consistency. Finger et al.13 reported WORQ to have a high level of internal consistency (Cronbachs alpha=0.88) and interlinker agreement (kappa=0.82). The WORQ also exhibit acceptable levels of test retest reliability (r=0.79) and good face, content and criterion validity." Use Vancouver style and do not use the names of the authors

 Rewrite the results section – report only results here.

Is the work clearly and accurately presented and does it cite the current literature?

No

If applicable, is the statistical analysis and its interpretation appropriate?

Partly

Are all the source data underlying the results available to ensure full reproducibility?

Partly

Is the study design appropriate and is the work technically sound?

Yes

Are the conclusions drawn adequately supported by the results?

No

Are sufficient details of methods and analysis provided to allow replication by others?

No

Reviewer Expertise:

Cardiac rehabiltiation. vocational rehabilitation

I confirm that I have read this submission and believe that I have an appropriate level of expertise to state that I do not consider it to be of an acceptable scientific standard, for reasons outlined above.

# Response

**Published:** 2023-11-10

**Authors:** Shaheed Soeker

**Affiliations:** University of the Western Cape, South Africa

**Response to reviewers**

 Dear Professor

 Thank you so much for the informed comments that you provided.

 I have provided a detailed response to the comments that you have provided. As a team we have tried to incorporate as many of the suggestions that you provided.

 Reviewer 2: **Thomas Maribo**, University of Copenhagen, Aarhus University, Blegdamsvej, Copenhagen, Denmark 

**Comment**

 Headings should align with a scientific paper and headings like “description of dependent and independent variable”, “Univariate/descriptive statistics”, and “bivariate analysis” should be removed.

**Response**

 Unnecessary headings have been removed

**Comment**

 Procedure starts with "The pilot study" – which pilot study? If I understand correct you could start with "A pilot study".

**Response**

 This has been changed to ‘A’ pilot study

**Comment**

 In the methods section: place the statistical programme at the end, not the beginning.

**Response**

 This has been removed from the beginning of the section.

**Comment**

 Delete redundant sentences like “The IBM SPSS software package is a well-established program used for batched and non-batched statistical analysis within health sciences research.” (this is a scientific paper not a text book).

**Response**

 Sentence has been removed.

**Comment**

 In the methods section, the impairment score is mentioned in 2 or 3 places. Rewrite and describe the outcome measures and then the analyses.

**Response**

 This question has been answered, the outcome measure was described first and then the analysis procedure as it relates to the study was described

**Comment**

 The term “the researcher” is used far too much (8 times!). The scientific world does not care what and how the researcher did - it is the study that is important. Rephrase “The researcher liaised with the head of department of internal medicine” - and more like that. And remove "The researcher and the researcher’s supervisor were satisfied" we do not care what your supervisor think or do. We want science!

**Response**

 The word ‘researcher’ has been reduced.

 Suggested removal of sentence done.

**Comment**

 Rewrite and shorten "Ethics". This is far too long with redundant sentences like "The WHO14 ethical guidelines help promote ethical conduct of research, through the enhancement and protection of the rights of the research participants and communities" – this is a scientific paper! "Participation was voluntary and participants were allowed to withdraw from the study at any stage." is redundant as is it already stated.

**Response**

 This section has been shortened and reformatted.

**Comment**

 The results section should start with a description of the population: how many patients were enrolled in CR, how many completed, how many (and who) did not complete CR – and how many did not complete the questionnaire

**Response**

 Description of the sample has been moved from methods to results section. As well as information related to the number of participants completing surveys, etc.

**Comment**

 The sentence "Logistic regression was performed to determine the participant’s ability to RTW and each of their socio-demographic, health and work characteristics were analysed using Pearson Chi-square test, with a significant p-value that is equal to or lower than 0.05." is placed in the results section! move to the methods section. If you want other researchers to read your paper it must be rewritten. I urge you to consult an experienced scientist.

**Response**

 Methods and result information have been separated into the appropriate sections.

**Comment**

 Rewrite the discussion and include the following:

- why did you use interview and not the questionnaire? What did that mean?
- Why were interviews held at the first authors private place? And could this bias the result?

**Response**

 Some participants required assistance with reading or comprehension therefore interviews were done. The article describes that educations levels varied which contributed to the level of understanding. 

 Telephonic interviews that were recorded were done from the researchers practice as it was logistically easier.

**Comment**

 Reread and check for abbreviations. cardiac rehabilitation (CR) is abbreviated in the 1st paragraph, yet cardiac rehabilitation is written out 3 times.

**Response**

 Abbreviations throughout have been revised.

**Comment**

 Rewrite " In the study conducted by Finger, Escorpizo, Bostan and De Bie,13 to identify the psychometric evidence for the use of the WORQ as a valuable instrument, they concluded that the WORQ showed a high test-retest reliability and good internal consistency. Finger et al.13 reported WORQ to have a high level of internal consistency (Cronbachs alpha=0.88) and interlinker agreement (kappa=0.82). The WORQ also exhibit acceptable levels of test retest reliability (r=0.79) and good face, content and criterion validity." Use Vancouver style and do not use the names of the authors

**Response**

 Sentence has been reworded and referenced without the authors name.

**Comment**

 Rewrite the results section – report only results here.

**Response**

 Result section has been rewritten as suggested.
